# Long noncoding RNA TPT1-AS1 promotes the progression and metastasis of colorectal cancer by upregulating the TPT1-mediated FAK and JAK-STAT3 signalling pathways

**DOI:** 10.18632/aging.202339

**Published:** 2021-01-10

**Authors:** Leiyi Zhang, Fei Ye, Zhongkun Zuo, Ding Cao, Yu Peng, Zedong Li, Jiangsheng Huang, Lunxi Duan

**Affiliations:** 1 Department of Minimally Invasive Surgery, The Second Xiangya Hospital of Central South University, Changsha 410011, Hunan, China

**Keywords:** colorectal cancer, long noncoding RNA, tumour protein translationally controlled 1 antisense RNA 1, TPT1, metastasis

## Abstract

Tumour protein translationally controlled 1 (TPT1) antisense RNA 1 (TPT1-AS1) is known to be involved in the development and metastasis of cervical and ovarian cancers; however, its biological role in colorectal cancer (CRC) remains unknown. This study aimed to determine the function and mechanism of action of TPT1-AS1 in the progression and metastasis of CRC. Elevated TPT1-AS1 levels were observed in CRC tissues. Furthermore, the high expression levels were found to be correlated with unfavourable clinicopathological characteristics in CRC. Cell function experiments demonstrated that TPT1-AS1 depletion impeded cell proliferation, migration and invasion and enhanced cell adhesion; it also attenuated tumorigenesis and metastasis *in vivo*. Additionally, TPT1-AS1 was predominately located in the nuclei of the cells and could upregulate the expression of TPT1 by recruiting mixed lineage leukaemia protein-1 (MLL1), which increased the trimethylation of H3K4 me3 in the TPT1 promoter region and subsequently activated FAK and JAK-STAT3 signalling cascades. The inhibition of FAK activation by PF573228 significantly attenuated the oncogenic effect of TPT1-AS1. These findings indicated that TPT1-AS1 promoted tumour progression and metastasis in CRC by upregulating TPT1 levels and activating the FAK and JAK-STAT3 signalling pathways. Thus, TPT1-AS1 may be considered as a potential therapeutic target for CRC.

## INTRODUCTION

Colorectal cancer (CRC) is the third most prevalent cancer with nearly 1.3 million new patients and more than 0.6 million deaths across the world every year [1]. Although notable advances in the early diagnosis and intervention have been made in recent years, the prognosis of CRC remains dismal as over half of the patients with advanced-stage cancer die due to recurrence and metastasis [2]. Therefore, the potential mechanisms involved in the development and progression of CRCs need to be identified.

Long noncoding RNAs (lncRNAs) belong to a subgroup of transcripts with lengths that exceed 200 nucleotides. Despite the presence of a protein-coding deficiency, lncRNAs are known to be versatile molecules that participate in various diseases, especially in the development and progression of tumours [3–5]. The mechanism of action of lncRNAs is extremely complicated and is mainly reflected in the regulation of the expression of the gene at the transcriptional and post-transcriptional levels and during epigenetic modification [6–8]. Numerous studies have verified that lncRNAs contribute to tumour progression, metastasis and recurrence in CRCs. For example, lncRNA CASC9 may enhances TGFβ2 mRNA stability and elevates the expression levels of TGFβ2 and TERT, leading to an increase in SMAD3 phosphorylation and TGF-β pathway activation and ultimately promoting CRC cell growth [9]. SNHG14 was found to contribute to CRC growth and metastasis by recruiting methyltransferase enhancer of zeste homolog 2 (EZH2), which could repress EPHA7 transcription via methylated modification [10]. GLCC1 was reported to be upregulated in CRC cells that were under glucose starvation, which might have contributed to cell survival by enhancing glycolysis [11]. Hence, exploring the potential mechanisms of action of lncRNAs in CRC might prove valuable in providing new strategies for the diagnosis and treatment of cancer.

Tumour protein translationally controlled 1 (TPT1) antisense RNA 1 (TPT1-AS1) is the transcript of the TPT1 gene located at the human locus of 13q14.13. Previous studies have reported the dysregulation of TPT1-AS1 in anaplastic glioma, cervical cancer and ovarian cancer [12–14]. In glioma, the expression level of TPT1-AS1 was downregulated with tumour grade; in addition, it was found to have a prognostic value for anaplastic gliomas [12]. On the contrary, TPT1-AS1 was upregulated in cervical and ovarian cancers and contributed to both tumorigenesis and metastasis [13, 14]. Nonetheless, this transcript has not been well characterised in malignancies, particularly CRC. Hence, this study aims to determine the function and mechanism of action of TPT1-AS1 in the development and progression of CRCs.

Here, we found that TPT1-AS1 was elevated in CRC tissues and cell lines, and the high expression level was associated with the clinicopathological features and poor prognosis in CRC patients. Based on the results of the *in vivo* and *in vitro* experiments, TPT1-AS1 was confirmed to facilitate cell proliferation, migration and invasion. Furthermore, TPT1-AS1 was found to upregulate TPT1 expression by recruiting the histone methyltransferase MLL1 to its promoter and increasing the trimethylation of H3K4 me3. TPT1 is required for the oncogenic effect of TPT1-AS1 in CRC. Our findings suggested that TPT1-AS1 promoted the proliferation, migration and invasion of the CRC cells via the activation of the focal adhesion kinase (FAK) and JAK-STAT3 signalling pathways. This study unearthed novel insights into the mechanism of action of TPT1-AS1, thus indicating its potential as an effective therapeutic target for CRC.

## RESULTS

### TPT1-AS1 is elevated in CRC tissues and its upregualtion predicts poor survival of CRC patients

**Figure 1:**
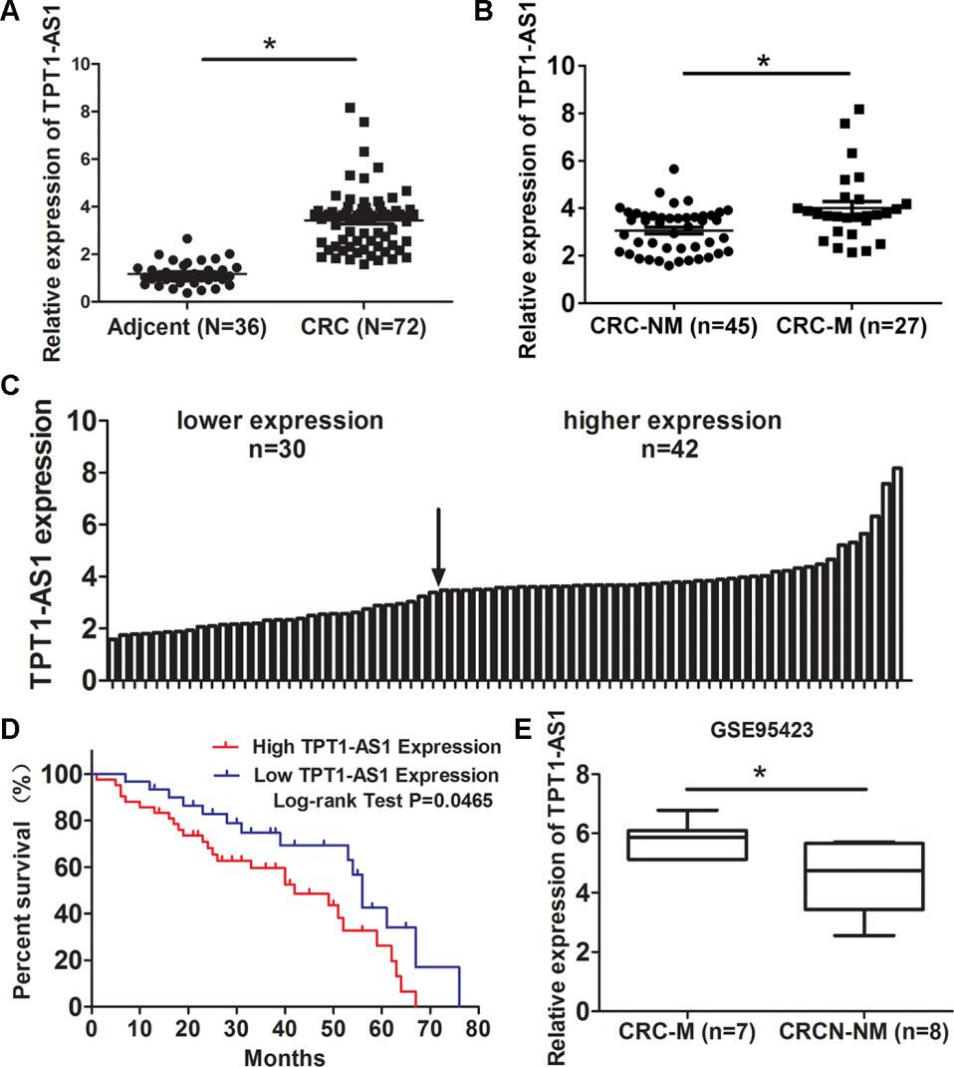
**TPT1-AS1 is elevated in CRC tissues and its upregualtion predicts poor survival of CRC patients.** (**A**) qRT-PCR detected TPT1-AS1 expression in 72 CRC and 36 adjacent normal tissues; (**B**) Expression of TPT1-AS1 was detected in metastatic CRC (CRC-M) and nonmetastatic tumor tissues (CRC-NM); (**C**) TPT1-AS1 expression in CRC patients was divided into high expression and low expression groups according to the median value; (**D**) The overall survival of CRC patients was evaluated by Kaplan-Meier analysis; (**E**) TPT1-AS1 expression was analyzed in metastatic CRC (CRC-M) and nonmetastatic tumor tissues from GEO data (GSE95423). *P<0.05.

Firstly, we examined TPT1-AS1 expression in 72 CRC and 36 adjacent normal tissues by qRT-PCR method. The result showed that TPT1-AS1 was upregulated in CRC tissues (Figure 1A). Moreover, The level of TPT1-AS1 in metastasis CRC tissues was significantly higher than nonmetastatic tumor tissues (Figure 1B). Base on the median value of TPT1-AS1 expression in tumor tissues, the CRC patients were assigned high expression group (n=42) and low expression group (n=30) (Figure 1C). The correlation of TPT1-AS1 expression and CRC clinical pathologic features were investigated by χ^2^ tests. As shown in Table 1, TPT1-AS1 expression was significantly correlated with tumor invasion depth (*P*=0.038), TNM stage (*P*=0.025) and distant metastasis (*P*=0.031). Additionally, the high TPT1-AS1 expression in CRC patients was implied the poor overall survival (Figure 1D). Besides, our result was consistent with the GEO data (GSE95423) which demonstrated TPT1-AS1 upregulation in CRC tissue with liver mmetastasis comparing to CRC tissue without metastasis (Figure 1E).

**Table 1:** The association between TPT1-AS1 expression and clinical pathology features.

**Variable**	**Cases**	**TPT1-AS1 expression**		***P* Value**	**χ2 Value**
		**Low**	**High**		
Age				0.194	1.264
<60	22	7	15		
≥60	50	23	27		
Gender				0.237	0.914
Male	36	17	19		
Female	36	13	23		
Tumor location				0101	2.299
Rectum	34	11	23		
Colon	38	19	19		
Tumor Size				0.149	1.646
<5	32	16	16		
≥5	40	14	26		
Tumor invasion depth				0.038	4.114
T1-2	24	14	10		
T3-4	48	16	32		
TNM stage				0.025	4.761
I+II	42	22	20		
III+IV	30	8	22		
Distance Metastasis				0.031	4.404
No	45	23	22		
Yes	27	7	20		

### TPT1-AS1 promotes CRC cell proliferation, migration and invasion *in vitro* and *in vivo*

**Figure 2:**
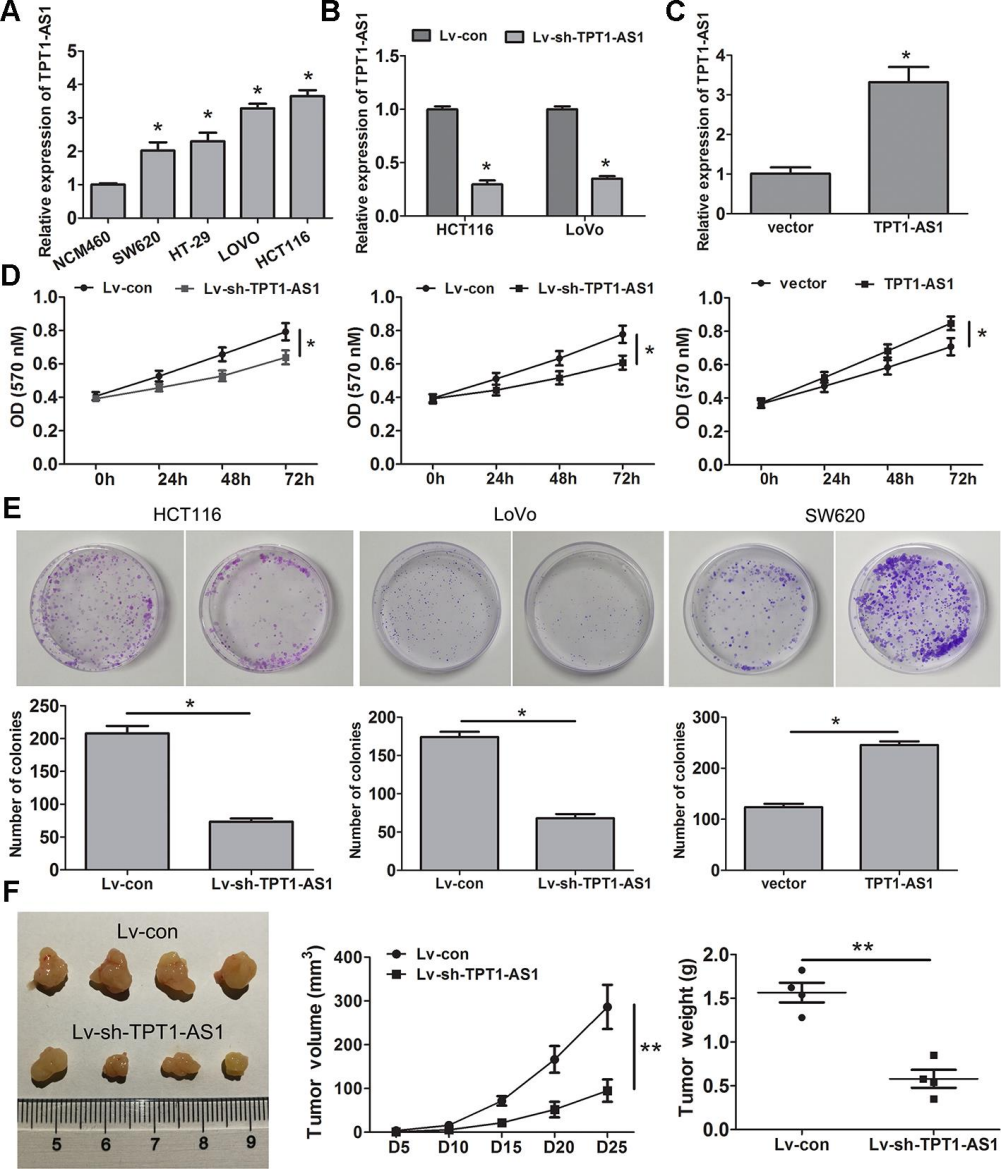
**TPT1-AS1 promotes CRC cell proliferation *in vitro* and *vivo*.** (**A**) TPT1-AS1 endogenous level in CRC cell lines and normal colonic cell line NCM460 was detected by qRT-PCR. TPT1-AS1 expression was examined in TPT1-AS1 silencing HCT116 and LoVo cells (**B**) and overexpressing SW620cells (**C**). MTT (**D**) and clone formation assays (**E**) were applied to assess the effect of TPT1-AS1 on CRC cell proliferation and growth. (**F**) TPT1-AS1 knockdown inhibited the tumor volume and weight in nude mice tumorigenicity assay. *P<0.05, **P<0.01.

To explore the role of TPT1-AS1 in CRC cells, the endogenous expression of this transcript in CRC cell lines (HCT116, HT-29, SW620 and LoVo) and a normal colonic cell line (NCM460) was detected by qRT-PCR. The result showed that the level of TPT1-AS1 was significantly elevated in the tumour cell lines, particularly HCT116 and LoVo cells, compared to that in the NCM460 cells (Figure 2A). Subsequently, TPT1-AS1 was knocked down in the HCT116 and LoVo cells and enhanced in the SW620 cells, and the levels were confirmed by qRT-PCR assay (Figure 2B, 2C, respectively). MTT and clone formation assays showed that TPT1-AS1 knockdown suppressed cell proliferation and growth, whereas TPT1-AS1 overexpression demonstrated promoting effects (Figure 2D, 2E). Importantly, the silencing of TPT1-AS1 significantly reduced tumour volume and weight (Figure 2F). This difference was further confirmed following the examination of the xenograft by H&E, the tumours that developed from TPT1-AS1-depleted cells displayed lower Ki-67 staining than those in the control group (Supplementary Figure 1). These results indicated that TPT1-AS1 exerted a significant promotive effect on the tumorigenesis of CRC *in vitro* and *in vivo*.

**Figure 3:**
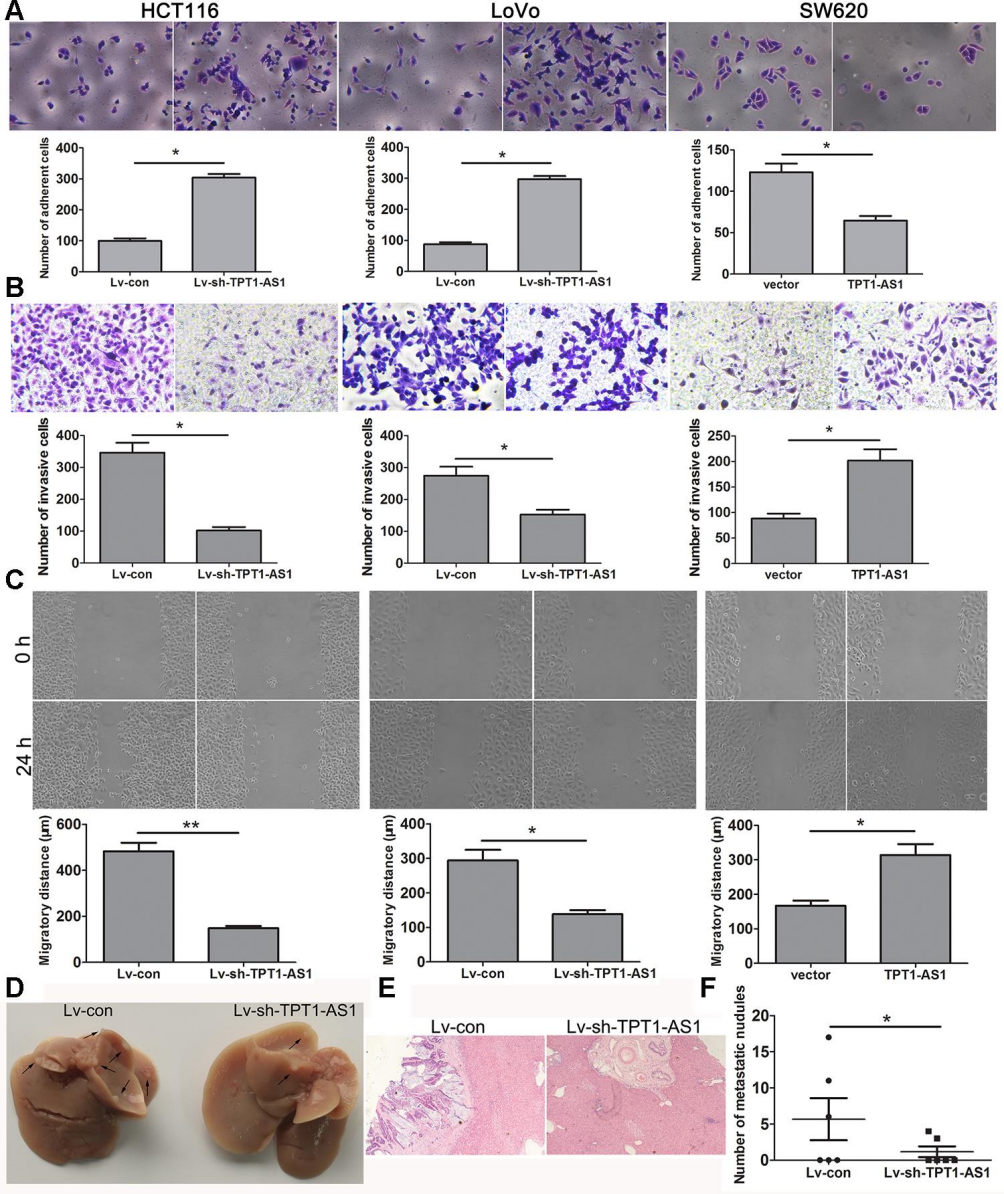
**TPT1-AS1 promotes CRC cell migration and invasion *in vitro* and *vivo*.** (**A**) Cell adhesion assay was applied to determine the effect of TPT1-AS1 on CRC cell adhesion (magnification 200x). Wound scratch (magnification 100x) (**B**) and Transwell assays (magnification 200x) (**C**) were performed to examine the effect of TPT1-AS1 on CRC cell invasion and migration. (**D**) TPT1-AS1 knockdown suppressed CRC liver metastasis *in vivo*, the gross specimen images of liver metastases. (**E**) The metastatic nodules in liver tissue were detected by HE staining (magnification 100x). (**F**) The statistical analysis of number of liver metastatic nodules. *P<0.05, **P <0.01.

As shown in Figure 3A, TPT1-AS1 knockdown significantly enhanced the cell adhesion ability, whereas TPT1-AS1 overexpression had the opposite effect. The migration and invasion assays demonstrated that TPT1-AS1 depletion significantly impaired cell migration and invasion, but the opposite was observed when TPT1-AS1 was overexpressed in the SW620 cells (Figure 3B, 3C). Furthermore, we investigated the effect of TPT1-AS1 on liver metastasis *in vivo*. As shown in Figure 3D–3F, the TPT1-AS1 knockdown group displayed a reduction in the number of metastatic modules in the liver when compared to the control group, indicating that TPT1-AS1 could promote cell migration and metastasis *in vitro* and *in vivo*.

### TPT1-AS1 upregulated the expression of TPT1 by H3K4 me3 modification

**Figure 4:**
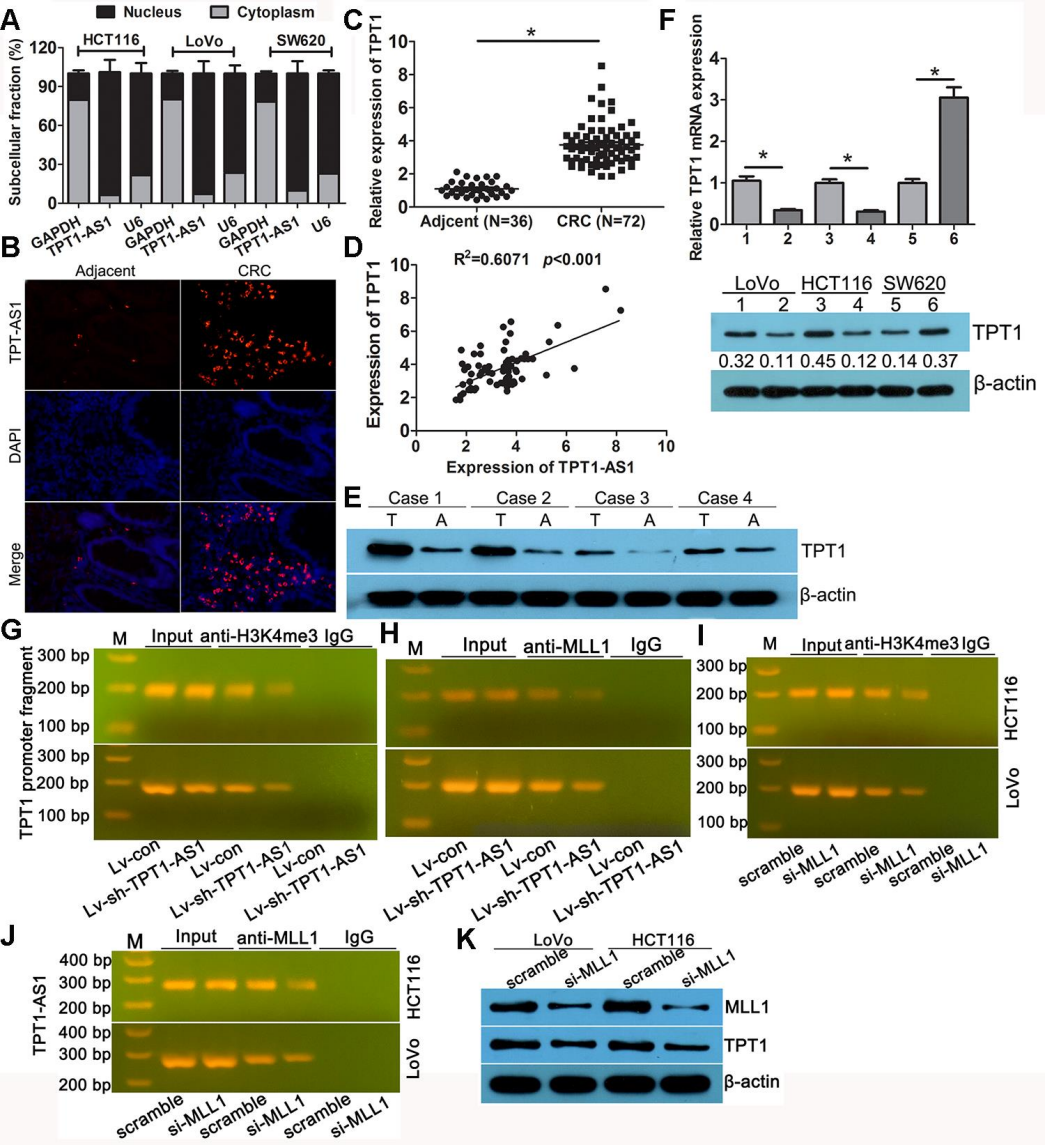
**TPT1-AS1 upregulated the expression of TPT1 by H3K4me3 modification.** (**A**) Subcellular fractionation analysis showed that TPT1-AS1was mainly enriched in the nucleus of CRC cells. (**B**) FISH analysis the subcellular localization of TPT1-AS1 in tissues (magnification 100x). (**C**) qRT-PCR detected TPT1 expression in 72 CRC and 36 adjacent normal tissues. (**D**) The correlation between TPT1-AS1 and TPT1 expression was analyzed in 72 CRC tissues. (**E**) Western blot showed that our CRC specimen had a obviously increased TPT1 level compared with adjacent normal tissues. (**F**) TPT1 mRNA (upper)and protein (down) level were examined in TPT1-AS1 overexpression and knockdown cells. (**G**) ChIP assay showed that TPT1-AS1 depletion reduced the H3K4me3 modification in TPT1 transcription region. (**H**) ChIP analysis reveals that MLL1 mediated TPT1-AS1 regulation TPT1 transcription. (**I**) ChIP analysis demonstrated that H3K4me3 level was reduced in the TPT1 promoter region after MLL1 depletion. (**J**) RIP assays confirmed that TPT1-AS1 could physically bind to MLL1. (**K**) TPT1 expression was impeded when MLL1 was silenced by siRNA. 1,3 stand for Lv-con group; 2,4 stand for Lv-sh-TPT1-AS1 group; 5 stand for vector group; 6 stand for TPT1 overexpression group. *P<0.05.

Subcellular fractionation analysis showed that TPT1-AS1 was mainly enriched in the nucleus of the HCT116, LoVo and SW620 cells (Figure 4A). The FISH analysis confirmed a similar subcellular localisation of TPT1-AS1 in the tissues (Figure 4B); moreover, the percentage of positive signals in the CRC tissues was higher than that in the adjacent tissues. Antisense lncRNA tends to modulate the expression of its sense mRNA; therefore, we speculated that TPT1-AS1 may regulate the level of TPT1 in CRC. Firstly, the expression level of TPT1 was elevated in the CRC specimens as detected by qRT-PCR, which was consistent with the expression level of TPT1-AS1 in CRC samples (Figure 4C). Additionally, a positive correlation between TPT1 and TPT1-AS1 expression levels were observed in the CRC tissues (Figure 4D). Similarly, the result of the Western blot analysis showed that the expression level of TPT1 was significantly increased in the CRC specimens when compared to that in the adjacent normal tissues (Figure 4E). Furthermore, the mRNA and protein expression levels of TPT1 were significantly reduced in the TPT1-AS1-silenced cells and increased in the cells that overexpressed TPT1-AS1 (Figure 4F). These findings suggested that TPT1-AS1 could upregulate TPT1 expression.

To further investigate the potential mechanism of TPT1-AS1 in upregulating the expression of TPT1 in CRC, we analysed the extent of histone modification in the ENCODE data. The activated histone modification H3K4 m3 was enriched around the TPT1 promoter region (Supplementary Figure 2). Whether TPT1-AS1 could recruit methyltransferase MLL1, the charge of H3K4 trimethylation modification. RNA-Protein Interaction Prediction analysis displayed an interaction between TPT1-AS1 and MLL1 (Supplementary Figure 3). The ChIP assay was applied to verify the interacting by using the anti-H3K4 me3 and MLL1 antibodies. As shown in Figure 4G, the amplification of the TPT1 promoter fragment (TPT1-pro) was reduced in the precipitates of the TPT1-AS1-silenced cells, implying that TPT1-AS1 mediated the H3K4 me3 modification in the TPT1 transcription region. The ChIP assay using the MLL1 antibody displayed a similar reduction in TPT1-pro (Figure 4H), indicating that MLL1 participated in TPT1-AS1 regulation TPT1 transcription. In addition, it demonstrated that the level of H3K4 me3 was reduced in the TPT1 promoter region after MLL1 depletion (Figure 4I). The RIP assays confirmed that TPT1-AS1 could physically bind to MLL1 (Figure 4J). TPT1 expression was impeded following the knockdown of MLL1 by siRNA (Figure 4K). Taken together, these results indicated that TPT1-AS1 recruited MLL1 to the promoter region of TPT1 and enhanced the H3K4 me3 level, resulting in the upregulation of TPT1.

### TPT1-AS1 promotes CRC progression via TPT1

**Figure 5:**
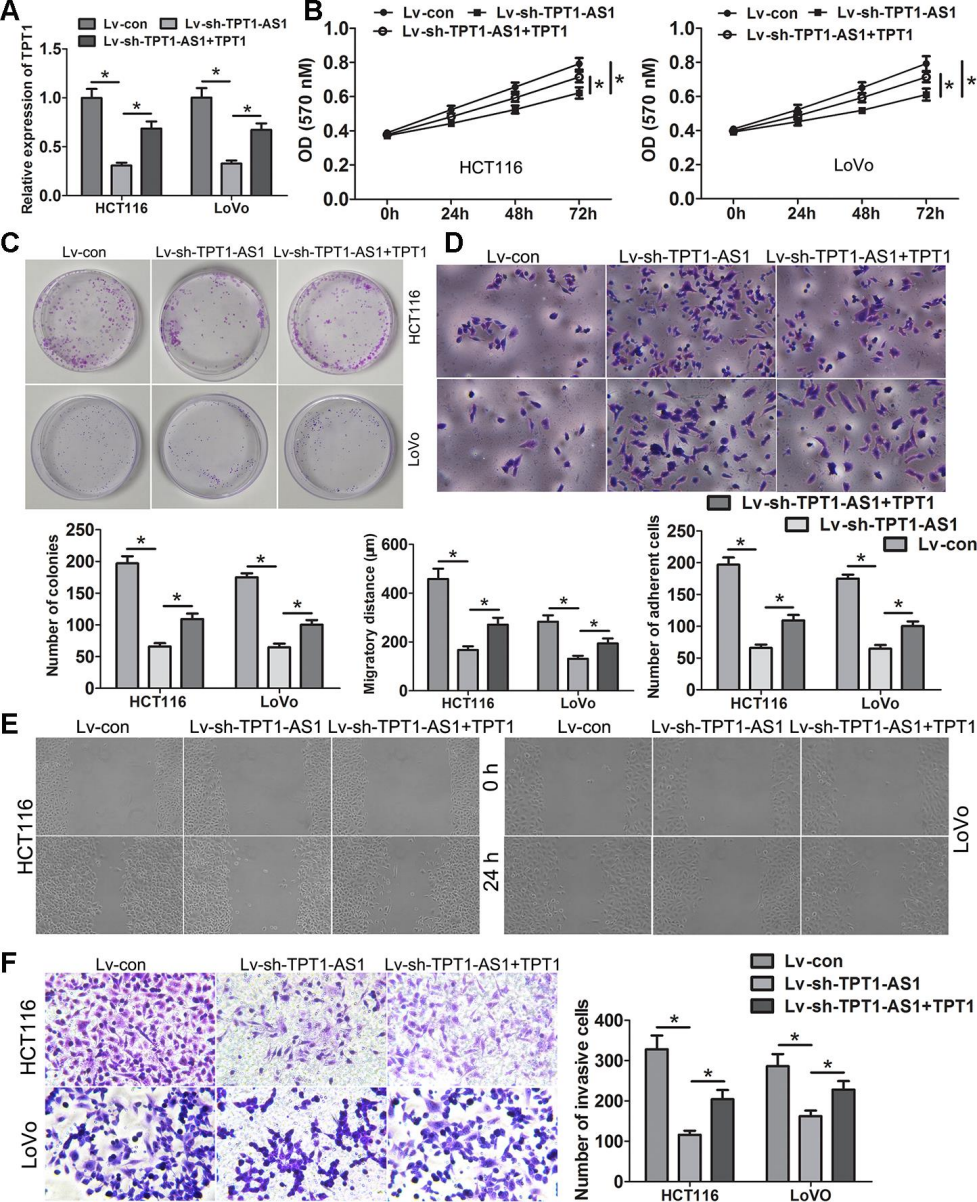
**TPT1-AS1 promotes CRC progression via TPT1.** (**A**) TPT1 expression was restored in TPT1-AS1 depletion cells via transfecting the pcDNA3.1-TPT1 plasmid. MTT (**B**) and clone formation assays (**C**) showed that ectopic expression of TPT1 could remarkably relieved the suppressive effects on cell viability and proliferation causing by TPT1-AS1 knockdown. (**D**) Restoring TPT1 expression could obviously attenuated the promotive effects on cell adhesion inducing by TPT1-AS1 depletion (magnification 200x). Wound scratch (magnification 100x) (**E**) and Transwell assays (magnification 200x) (**F**) showed that ectopic expression of TPT1 could obviously relieved the suppressive effects on cell migration and invasion causing by TPT1-AS1 knockdown. *P<0.05.

To evaluate the role of TPT1 on the TPT1-AS1-mediated increase in cell proliferation, migration and invasion in the CRC cells, rescue experiments were performed. The expression level of TPT1 was elevated in the TPT1-AS1-silenced cells by transfecting them with a pcDNA3.1-TPT1 plasmid and verified by qRT-PCR (Figure 5A). As shown in the functional experiments, the ectopic expression of TPT1 significantly reduced the suppressive effects on cell proliferation, migration and invasion and attenuated the promotive effects on cell adhesion mediated by TPT1-AS1 knockdown (Figure 5B–5F).

### TPT1-AS1 promoted CRC progression via TPT1/FAK/JAK-STAT3 signalling

**Figure 6:**
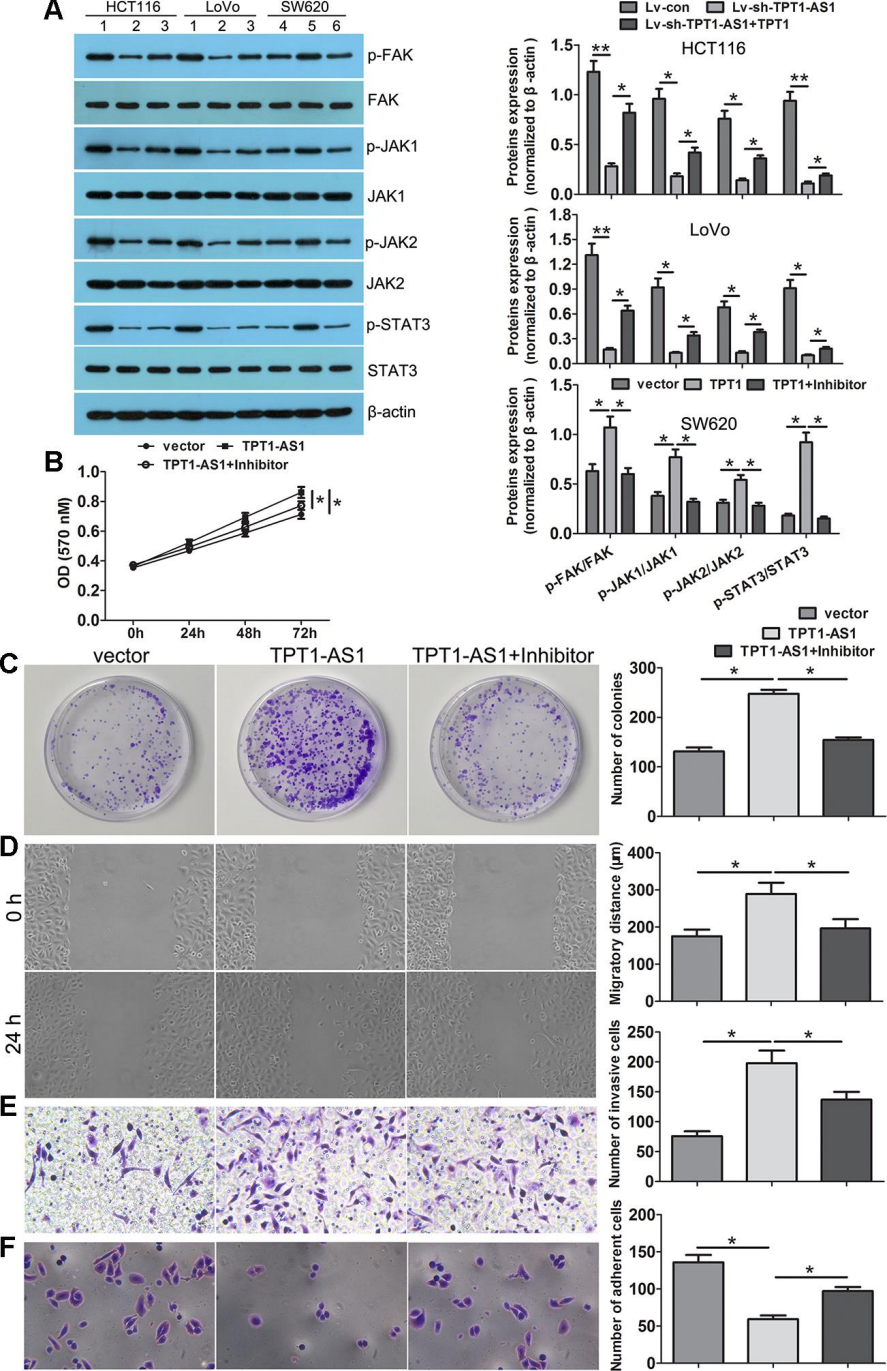
**TPT1-AS1 promoted CRC progression via the TPT1/FAK/JAK-STAT3 signaling.** (**A**) Western blot was conducted to examine p-FAK, FAK, p-JAK1, JAK1, p-JAK2, JAK2, p-STAT3 and STAT3 expression in TPT1-AS1 knockdown and overexpression cells. FAK inhibitor (PF573228) could remarkably attenuated the promotional effects on cell viability (**B**), colony formation (**C**), migration (magnification 100x) (**D**) and invasion (magnification 200x) (**E**), and inhibited adhesion ability (magnification 200x) (**F**) in TPT1-AS1-overexpressing SW620 cells. 1 stand for Lv-con group; 2 stand for Lv-sh-TPT1-AS1 group; 3 stand for Lv-sh-TPT1-AS1+TPT1 group; 4 stand for vector group; 5 stand for TPT1 overexpression group; 6 stand for TPT1+ FAK inhibitor group. *P<0.05, **P<0.01.

**Figure 7:**
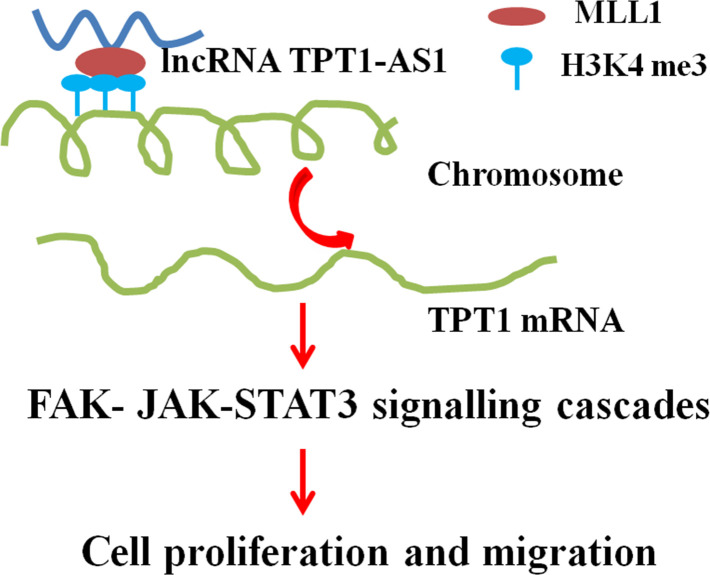
**A proposed model for illustrating the function and mechanism of TPT1-AS1 in CRC growth and metastasis.**

To investigate the underlying mechanisms of TPT1-AS1/TPT1 in CRC progression, we performed the GSEA analysis using the TCGA CRC datasets and found that TPT1 expression was positively correlated with FA and JAK-STAT3 signalling (Supplementary Figure 4). Interestingly, the positive correlation between TPT1- AS1 and FAK, JAK1, JAK2 and STAT3 expression levels were observed in the CRC tissues (Supplementary Figure 5). Western blot detection showed that the expression levels of p-FAK, p-JAK1, p-JAK2 and p-STAT3 were significantly reduced following TPT1-AS1 knockdown in the HCT116 and LoVo cells; however, this decrease was effectively mitigated when TPT1 was ectopically overexpressed. Alternatively, the levels of p-FAK, p-JAK1, p-JAK2 and p-STAT3 were increased in the TPT1-AS1-overexpressed SW620 cells (Figure 6A). Importantly, this increase was effectively attenuated when the cells were treated with the FAK inhibitor (PF573228). Furthermore, the FAK inhibitor significantly attenuated the promotional effects on cell proliferation, migration and invasion and enhanced the adhesion ability in the TPT1-AS1-overexpressed SW620 cells (Figure 6B–6F). These results indicated that TPT1-AS1 promoted CRC progression via the TPT1/FAK/JAK-STAT3 signalling pathway (Figure 7).

## DISCUSSION

Emerging studies have confirmed that numerous dysregulated lncRNAs are involved in the development and progression of CRC [15–17]. Therefore, exploring the function and molecular mechanisms of lncRNAs may aid in the identification of novel and valuable targets for the treatment of CRC. In this study, we demonstrated that the lncRNA TPT1-AS1 was upregulated in the CRC tissues and cell lines. The high expression of TPT1-AS1 was associated with unfavourable CRC clinicopathological characteristics such as advanced stage, lymph node metastasis and poor prognosis. Furthermore, TPT1-AS1 knockdown significantly inhibited cell proliferation, migration and invasion and enhanced cell adhesion. Moreover, TPT1-AS1 could upregulate TPT1 expression by recruiting MLL1 to the promoter region of TPT1 and enhances the H3K4 me3 level. The oncogenic effect of TPT1-AS1 on CRC cells and tissues was dependent on TPT1. In addition, TPT1-AS1 was found to promote the development and progression of CRC via the FAK and JAK-STAT3 signalling cascades.

The dysregulation of TPT1-AS1 has been reported in anaplastic glioma [12], cervical cancer [13] and ovarian cancer [14]; however, the expression and function of this lncRNA vary among these three tumour types. The expression level of TPT1-AS1 was decreased with the tumour grade and had prognostic value in anaplastic glioma. By predicting the target genes from the CLIP-seq data, it was speculated that TPT1-AS1 might act as a tumour suppressor [12]. However, in cervical cancer, the expression level of TPT1-AS1 was upregulated and associated with poor prognosis and overall survival [13]. Moreover, TPT1-AS1 was mainly distributed in the cytoplasm and promoted cell proliferation as well as metastasis by serving as a miR-324-5p sponge, thus implying its role as an oncogenic lncRNA in cervical cancer [13]. Similar results were observed in epithelial ovarian cancer (EOC), where TPT1-AS1 was upregulated in metastatic the tissues, and related to the adverse prognostic characteristics. The difference was that TPT1-AS1 was preferentially localised in the nuclei of the EOC cell, and the oncogenic roles of this lncRNA in tumour growth and metastasis were exerted via the positive regulation of both TPT1 and the downstream PI3K/AKT pathway [14]. In the present study, we confirmed that TPT1-AS1 facilitated cell proliferation, migration and invasion in CRC and a high expression level of TPT1-AS1 indicated poor prognosis and overall survival. These results were consistent with those observed in cervical and ovarian cancers.

Accumulated evidence has confirmed that a part of the lncRNAs take part in epigenetic regulation via DNA methylation and histone modifications (such as methylation and acetylation) [18]. Histone methylation usually occurs on the different lysine residues of histone H3/H4 and is mediated by histone methylases or demethylases [19]. LncRNAs act as decoys that recruit and bind with these relevant enzymes during histone modifications. The EZH2 is a subunit of the polycomb repressive complex 2 (PRC2) and has a catalytic activity, which can increase histone H3 lysine 27 trimethylation (H3K27 me3), resulting in chromatin compression and disturbances in gene transcription [20, 21]. Lysine-specific demethylase 1 (LSD1) is a demethylase that can erase mono- and dimethylated residues of histone H3 lysine 4 (H3K4 me1, H3K4 me2) and mono-residues of histone H3 lysine 9 (H3K9 me1), leading to the inhibition of the transcription [22]. Moreover, LSD1 was found to promote transcription following the demethylation of H3K9 me2 [23]. MLL1 can specifically induce H3K4 me3 and activate transcription [24, 25]. For instance, lncRNA HOXD-AS1, the antisense RNA 1 of the HOXD cluster, recruits PRC2, resulting in an increase in the level of H3K27 me3 and the suppression of HOXD3 transcription in CRC [26]. LncRNA CASC9 was reported to recruit EZH2 to the PDCD4 promoter and increase the H3K27 me3 level, causing a decrease in the expression level of PDCD4 in oesophageal squamous cell carcinoma [27]. In hepatocellular carcinoma (HCC), GAS8 antisense RNA 1 (GAS8-AS1) recruited H3K4 methyltransferase MLL1 to the promoter of GAS8 and enhanced the H3K4 m3 level, thereby upregulating the expression level of GAS8 [28]. In another study, FEZF1 antisense RNA 1 (FEZF1-AS1) recruited demethylase LSD1 to the promoter of p21 and reduced the H3K4 m2 level, resulting in the repression of p21 expression in gastric cancer [19]. In our study, the expression levels of TPT1-AS1 and TPT1 were positively correlated, and TPT1 expression was regulated by TPT1-AS1. Most importantly, TPT1-AS1 enhanced the H3K4 m3 level in the TPT1 promoter region by recruiting and binding to MLL1, thus altering the chromatin status from inactive to active and ultimately promoting TPT1 transcription. We first elucidated TPT1-AS1 regulating TPT1 expression from an epigenetic perspective.

TPT1 is a highly conservative multifunctional protein and is involved in multiple physiological activities such as cell growth, cell proliferation and metabolism [29]. Moreover, TPT1 was verified as a crucial factor in cancer reversion [30]. It was found to be upregulated in various cancers, including breast cancer, pancreatic cancer, HCC and EOC [14, 31–33]; furthermore, the high expression of TPT1 was significantly associated with the malignant behaviour of the tumour and the prognosis of the patient. In CRC, TPT1 was a potential diagnostic marker involved in the progression and metastasis of the disease. The high expression of the lncRNA was associated with the unfavourable clinicopathological characteristics of CRC. Moreover, TPT1 facilitated the migration and invasion of the cells and distant metastasis in the liver [34]. TPT1 significantly relieved the inhibitory effects of TPT1-AS1 knockdown on cell proliferation, colony formation, migration and invasion in CRC, thus indicating the oncogenic TPT1-dependent role of TPT1-AS1 on tumorigenesis and progression of CRC. These results were consistent with that reported in EOCs.

Metastasis is one of the causes of the high rates of tumour mortality. Cell migration and invasion are considered as the critical steps for cancer metastasis, and these processes are characterised by rearrangement of the cytoskeleton and abnormal cell adhesion [35]. Focal adhesions (FAs) are large, dynamic protein complexes that link the cytoskeleton to the extracellular matrix and play a pivotal role in cell migration [36]. FAK is a non-receptor tyrosine kinase that is localised within the cellular focal adhesions and mediated FA turnover [37]. Previous studies have shown that repression of FAK activity may contribute to CRC therapy [38, 39]. JAK/STAT3 is a well-characterised oncogenic signalling pathway and is pivotal in promoting carcinogenesis [40]. Numerous studies have confirmed that the JAK/STAT3 pathway is aberrantly activated in CRC [41–43]. Our GSEA analysis displayed that the high expression of TPT1 was associated with FA and JAK-STAT3 signalling pathway. FAK phosphorylation was reported to promote STAT3 activation and MMP-2 activity in gliomas [44]. In the current study, TPT1-AS1/TPT1 promoted cell migration and invasion by activating the FAK/STAT3 signalling cascades. In addition, the repression of FAK activation remarkably impeded JAK/STAT3 signalling and mitigated the promoting effects on CRC cell proliferation, colony formation, migration and invasion caused by TPT1-AS1 overexpression. These findings suggested that TPT1-AS1/TPT1 promotes tumorigenesis and progression of CRC via the FAK and JAK-STAT signalling cascades.

## CONCLUSIONS

The present study demonstrated that TPT1-AS1 promotes the progression and metastasis of CRC by upregulating the TPT1 expression and activating the FAK and JAK-STAT3 signalling pathways. Thus, TPT1-AS1 might be used as a potential therapeutic target for CRC.

## MATERIALS AND METHODS

### Clinical specimens

A total of 72 CRC tissues and 36 adjacent tissues were enrolled from the second Xiangya Hospital, Central South University, from March 2012 to April 2016. No patient was administered to preoperative chemotherapy and radiotherapy. The fresh tissues were fast frozen in liquid nitrogen and kept at -80° C. All of the patients were followed-up regularly intervals after surgery. This study was approved by the Ethics Committee of the Second Xiangya Hospital of Central South University and informed consent have signed by all patients. The pathological features were acquired from patients’ medical records.

### Cell culture and transfection

The CRC cell lines (HCT116, LoVo, SW620 and HT-29) and normal colonic epithelial cell line NCM460 were purchased from American Typer Culture Collection (ATCC) and cultured according to the instructions.

TPT1-AS1 siRNAs specially targeting TPT1-AS1 were designed and synthesized by GenePharma (Shanghai, China). The most interference effectiveness of target sequence was chosen to be packaged lentiviruses by GenePharma (Shanghai, China). A scrambled was used as negative control. To construct TPT1-AS1 downregulated cells model, HCT116 or LoVo (1×10^5^ cells) were mixed with polybrene (5 μg/ml), and Lv-sh-TPT1-AS1(4×10^8^ TU/ml, 5 μL), or Lv-con(2×10^8^ TU/ml, 10 μL). 48 hours later, transfected cells were selected by 20 μg/ml puromycin (Thermo Fisher, USA).

For overexpressional plasmids construction, the TPT1-AS1 sequence and TPT1 cDNA ORF were synthesized and subcloned into the pcDNA3.1 vector (General Biosystems, Chuzhou, China). Plasmids were transfected into cells using Lipofectamine 2000 (Thermo Fisher, USA) following the manufacturers’ instructions. Cells were harvested at 48 h after transfection.

### qRT-PCR

Total RNAs were isolated with Trizol (Thermo Fisher, USA). cDNA was reversely transcribed with RevertAid First Strand cDNA Synthesis Kit (Thermo Fisher, USA) following the instructions. qRT-PCR was conducted with SYBR Green I (TOYOBO, Japan) and detected by 7500 Real-Time PCR System (ABI, USA). Internal control was chosen β-actin gene for normalizing. qRT-PCR primers sequences were listed as following: TPT1-AS1 forward: 5’- GGTCAGCTCCAAGGAGGCTAT-3’, TPT1-AS1 reverse: 5’- GCCAGTGCTCTGAAGGAAAAC’; TPT1 forward: 5’- CGAGTTTCAGGCTCGTGCTA -3’, TPT1 reverse: 5’-TTCCTTCCTGGGCATGGAGTC-3’; β-actin forward: 5’-TGGCATCCACGAAACTACCT-3’, β-actin reverse: 5’-TCTTCATTGTGCTGGGTGCC-3’.

### MTT and Clone formation assays

Cell viability and proliferation were examined by MTT and clone formation assays respectively. For MTT detection, each group cells (1×10^4^ cells/well) were seeded into 96-well plates. 24 h later, each well was added 20 μl MTT and incubated at 37° C for 4 h. After removing the culture, each well was supplemented 150 μl of DMSO and incubated at 37° C for 10 min. Subsequently, the absorbance was detected at 570 nm by the microplate reader Thermo K3 (Thermo Scientific, USA). For clone formation experiment, 1×10^3^ cells were plated into a 35 mm dish and cultured for nearly two weeks. Then, colonies of cells were fixed in 4% paraformaldehyde (PFA) and stained with 0.01% crystal violet dye.

### Cell adhesion analysis

Cells (5×10^4^ cells/well) were seeded in 24-well plate coated with 1:5 diluted Matrigel (Corning, USA), and incubated at 37° C for 30 min. Then removing the culture and non- adhered cells, the adhered cells were rinsed by PBS twice times and fixed in 4% PFA for 30 minutes. Subsequently, cells were stained with 0.1% crystal violet for 20 minutes at room temperature.

### Cell migration and invasion analysis

Wound scratch was applied to examine cell migration. Simply, cells were plated in the 12-well plates and grown to 100% confluence. Cell wounds were scratched using a 20 μl pipette tube. Wound closure was measured the distance between the opposite edges of the wound after 0 and 24 h. BioCoat Matrigel Invasion chamber (Corning, USA) was applied to invasion assay. 1×10^5^ cells suspended in serum-free medium were plated into the upper chambers (coated with matrigel), while the lower chambers were added medium containing 10% FBS. Incubating at 37° C for 48 h, the translocated cells were fixed with 4% PFA and stained by 0.1% crystal violet for 20 min. Subsequently, the invasive cells were photographed and counted under the microscope.

### Western blotting

RIPA buffer containing protease and phosphatase inhibitors was used to lyse cells on ice for 0.5 h using. Proteins were extracted from supernatant of cell lysate and quantified the concentration by BCA Kit (Thermo Fisher, USA). 30 μg proteins per sample were separated by 10% SDS-PAGE and transferred to a polyvinylidene difluoride (PVDF) membrane (Thermo Fisher, USA). The membrane were blocked by 5% non-fat dry milk, and then added primary antibodies against TPT1 (1:1000, Abcam), p-FAK (Try397) (1:1000, CST), FAK (1:1000, CST), p-JAK1(1:1000, Abcam), p-JAK1 (1:1000, Abcam), p-JAK2 (1:1000, CST), JAK2 (1:1000, CST), p-STAT3 (1:1000, CST), STAT3 (1:1000, CST), overnight at 4° C, followed by incubating with HRP-labeled secondary antibody. The blotting signal was examined by using enhanced chemiluminescence reagent (Thermo Fisher, USA). β-actin used as control.

### Subcellular fractionation

The nuclear and cytosolic fractions of LoVo and SW620 cells were separated with PARIS Kit (Thermo Fisher, USA) following the manufacturers’ instructions.

### Fluorescence in situ hybridization (FISH)

The probe of TPT1-AS1 was designed and synthesized by BersinBio (Guangzhou, China), and its sequence was 5’-TTGGAGCTGACCTGAAGTGAAGATCTGGGAGTGG-3’ and labeled with CY3. FISH was conducted according to previously described methods [27]. Fresh tissue samples were fixed with 4% PFA for 48 h and dehydrated using graded ethanol. After vitrification by dimethylbenzene and embedding in paraffin, the tissues were subjected to dewaxing and hydration. After denatured at 73° C for 3 min, the sections were covered with hybridization solution that containing 5 ng/μl probes. Hybridization was placed in a moist chamber at 42° C overnight. Then the sections were rinsed with 4× Saline Sodium Citrate (SSC) containing 1% Tween-20, descending series of SSC for 5 min at 42° C. Subsequently, the nucleus was stained by DAPI. The sections were observed and photographed by fluorescent microscopy (Nikon, Japan).

### Chromatin Immunoprecipitation (ChIP)

ChIP experiment was conducted by the EZ-Magna ChIP kit (Millipore, USA) and carried out as previous described [27]. Cells were fixed in 4% PFA and quenched with glycine for 10 min. DNA was broken into 200 to 600 bps through ultrasonication. The lysates were immunoprecipitated with anti-H3K4me3 antibody (ab213224, Abcam, USA) or anti-MLL1 (NB600-256, Novus, USA) or rabbit IgG. The ChIP product was analyzed by PCR. Primers used in ChIP assay were presented as following: TPT1-pro forward: 5’-AGCGGCTGAGTCGGCCTTTTC-3’, TPT1-pro reverse: 5’-TGTGCGGCAGTAAGGATAGTG-3’.

### RNA immunoprecipitation (RIP) assay

RIP was conducted by EZ-Magna RIP kit (Millipore, USA) and performed as previous study [27]. Simply, cells were crosslinked with 1% formaldehyde and lysed in RIPA buffer containing proteinase and RNase inhibitor. Then the magnetic beads pre-conjugated with anti-MLL1 antibody (NB600-256, Novus, USA) or IgG were incubated with the cell lysate at 4° C overnight. RNA was purified from immunoprecipitataion complex and RT-PCR was applied to assess the expression of TPT1-AS1. Primers were presented as following: TPT1-AS1 forward: 5’-GAGACACAAGGCTCCGTTCC-3’, TPT1-AS1 reverse: 5’- AACAGCCAGGTTTTGAGAGC-3’.

### Gene set enrichment analysis (GSEA)

The GSEA was launched to analyze gene sets correlated with TPT1 in CRC. Gene expression data of CRC were downloaded from TCGA database. TPT1 expression was set high and low categories base on the median expression value. The TPT1 correlated gene sets and pathways were explored in the c2.cp.kegg.v7.0.symbols.gmt data set by GSEA v3 soft. P <0.05 and false discovery rate (FDR) <0.25 was the criterion for identifying statistically enriched genes.

### *In vivo* tumorigenic and metastasis assays

4-week-old male BALB/c nude mice were fed according to the guidelines authorized by the Animal Care Committee of the second Xiangya Hospital, Central South University.

For vivo tumorigenicity, animals were randomly divided into two groups (n=5 for each group) and transplanted subcutaneously with 1×10^7^ HCT116-Lv-sh-TPT1-AS1 or HCT116-Lv-con at the right flank. Tumor growth was measured and recorded every 5 days. 25 days later, animals were killed after anesthetized and stripped the tumors. Tumor volume was calculated by the formula: (length × width^2^)/2. The tumors tissues were paraffin-embedded, formal infixed and performed H&E staining, immunostaining analysis for Ki-67 protein expression.

For metastasis assay, Lv-sh-TPT1-AS1 or control Lv-sh-con lentivirus infected HCT116 cells (5×10^6^/ 0.2 ml PBS) were intrasplenic injected into each mouse (n=6 for each group). 8 weeks later, animals were killed after anesthetized and liver tissues were surgically excised. The the liver tissues were fixed in formalin and embedded in paraffin for hematoxylin and eosin (HE) examination. Metastatic nodules were analyzed under microscopy.

### Statistical analysis

Each assay was performed at least three times, the data were presented as mean ± standard deviation (SD). The statistical analysis was conducted by SPSS 20.0 software (SPSS Inc., IL, USA). Student’s t-test was applied to analyze the differential expression between two groups. The χ^2^ test was applied to assess the relationship between TPT1-AS1 expression and clinicopathological features of CRC. Survival was estimated by the Kaplan-Meier method. *P* < 0.05 were identified statistically significant.

## Supplementary Material

Supplementary Figures
