# Supplementary material for: Long noncoding RNA TPT1-AS1 promotes the progression and metastasis of colorectal cancer by upregulating the TPT1-mediated FAK and JAK-STAT3 signalling pathways
Source: Aging (Albany NY). 2021 Jan 10;13(3):3779–97. doi: 10.18632/aging.202339 (PMC7906141; doi:10.18632/aging.202339)
Supplement: Supplementary Figures [file aging-13-202339-s001.pdf]

SUPPLEMENTARY FIGURES

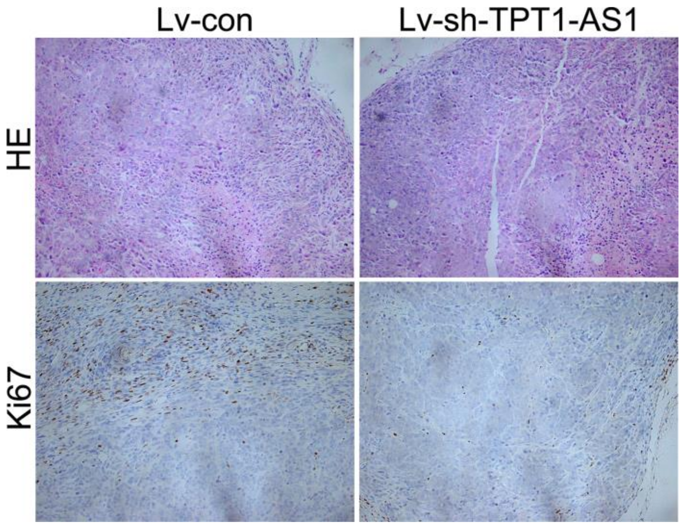

Supplementary Figure 1. The tumor sections were under H&E staining and IHC staining using antibody against Ki-67 (magnification 200x).

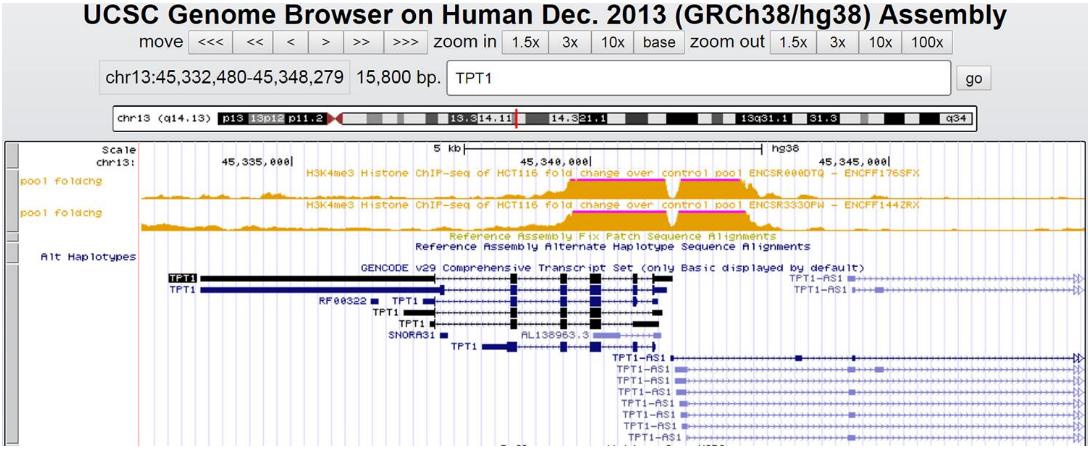

Supplementary Figure 2. ENCODE data analyzed the histone H3K4m3 modification of TPT1-AS1 promoter region.

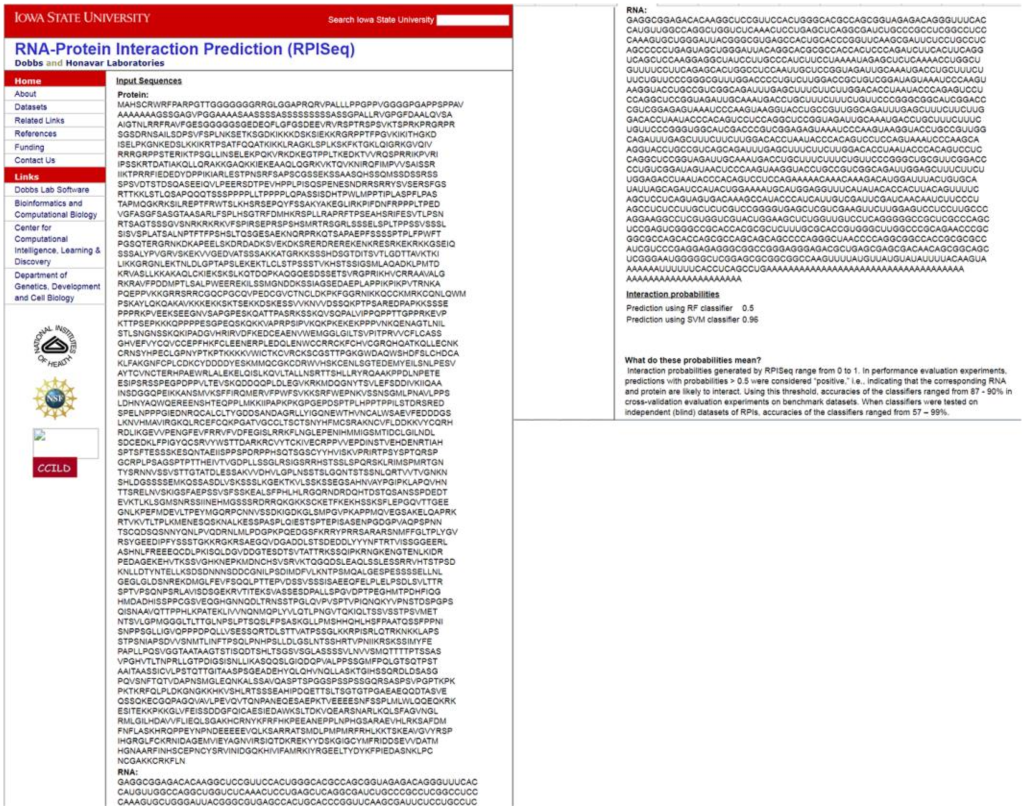

Supplementary Figure 3. RNA-Protein Interactuin Prediction (RPISeq) analysis displayed TPT1-AS1 interacting with MLL1.

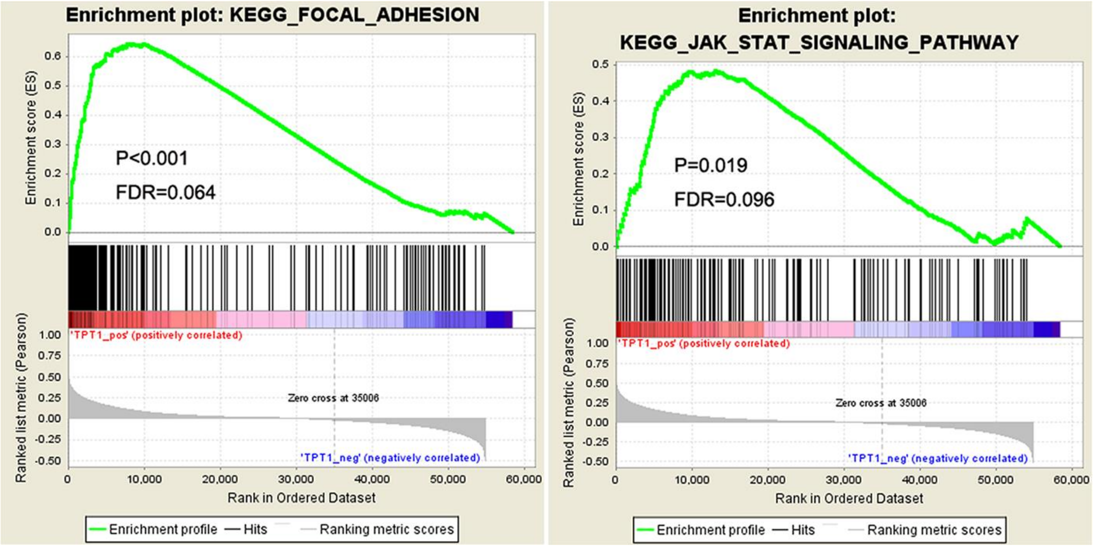

Supplementary Figure 4. GESA analysis found that TPT1 expression was positive correlation with focal adhesion and JAK-STAT signaling pathway.

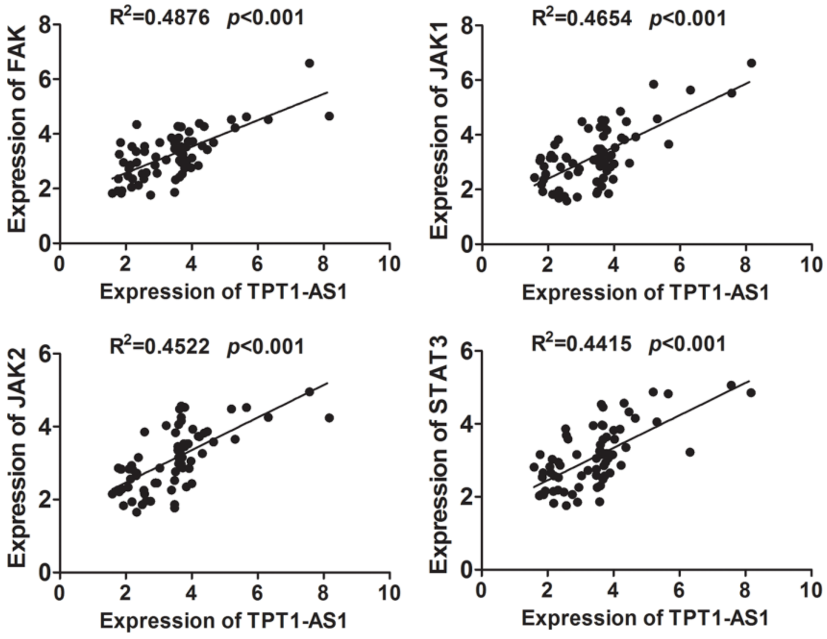

Supplementary Figure 5. The correlation between TPT1-AS1 and FAK, JAK1, JAK2 and STAT3 expression were analyzed in 72 CRC tissues.
